# Epidemiological characteristics, routine laboratory diagnosis, clinical signs and risk factors for hand, -foot -and -mouth disease: A systematic review and meta-analysis

**DOI:** 10.1371/journal.pone.0267716

**Published:** 2022-04-28

**Authors:** Zhijie Yi, Shujun Pei, Wenshuai Suo, Xiaoyang Wang, Zengyuan Huang, Aihua Yi, Bohao Wang, Zhiquan He, Ruolin Wang, Yi Li, Wei Fan, Xueyong Huang

**Affiliations:** 1 College of Public Health, Zhengzhou University, Zhengzhou, China; 2 College of Public Health, Xinxiang Medical University, Xinxiang, China; 3 Fifth Clinical Medical College of Henan University of Chinese Medicine, Zhengzhou, China; 4 First Affiliated Hospital of Shaoyang University, Shaoyang, China; 5 Quality Control Department of Henan Children’s Hospital, Zhengzhou, China; 6 Henan Province Center for Disease Control and Prevention, Zhengzhou, China; Meyer Children’s University Hospital - University of Florence, ITALY

## Abstract

**Background:**

For the past few years, only a few monovalent EV71 vaccines have been developed, while other enterovirus vaccines are in short supply. We conducted a quantitative meta-analysis to explore the epidemiological characteristics, routine laboratory diagnosis, clinical signs and risk factors for hand, foot and mouth disease (HFMD).

**Methods:**

PubMed, Embase and the Web of Science were searched for eligible reports published before April 16, 2021, with no publication time or language restrictions. The primary outcome was the odds ratio of the epidemiological characteristics, routine laboratory diagnosis, and clinical signs associated with HFMD severity and death.

**Results:**

After screening 10522 records, we included 32 articles comprising 781903 cases of hand, foot and mouth disease. Patients with severe illness developed some clinical signs (hypersomnia (OR = 21.97, 95% CI: 4.13 to 116.74), convulsion (OR = 16.18, 95% CI: 5.30 to 49.39), limb shaking (OR = 47.96, 95% CI: 15.17 to 151.67), and breathlessness (OR = 7.48, 95% CI: 1.90 to 29.40)) and had some changes in laboratory parameters (interleukin-6 levels standardized mean difference (SMD) = 1.57, 95%CI: 0.55 to 2.60), an increased neutrophils ratio (SMD = 0.55, 95%CI: 0.17 to 0.93), cluster of differentiation 4 (CD4+) (SMD = -1.38, 95%CI: -2.33 to -0.43) and a reduced lymphocytes ratio (SMD = -0.48, 95%CI: -0.93 to -0.33)) compared with patients with mild illness. The risk factors for death included cyanosis (OR = 5.82, 95% CI: 2.29 to 14.81), a fast heart rate (OR = 3.22, 95% CI: 1.65 to 6.30), vomiting (OR = 2.70, 95% CI: 1.33 to 5.49) and an increased WBC count (SMD = 0.60, 95% CI: 0.27 to 0.93).

**Conclusions:**

China has the highest incidence of HFMD. Our meta-analyses revealed important risk factors that are associated with the severity and mortality of HFMD.

## Introduction

Hand, foot and mouth disease (HFMD) is an infectious disease caused by enteroviruses, mainly enteroviruses 71 (EV71), coxsackievirus-A16 (CoxA16) and others, and usually affects children under five years old [1, 2]. The virus is generally spread via fecal–oral or personal contact with a sick person or their belongings. Oral pain, maculopapular rash, ulcers appearing on the oral mucosa, and emerging blisters on the hands, feet, and buttocks are typical symptoms of HFMD [3–5]. In recent years, HFMD outbreaks have been found in several parts of the world, especially in countries in the Asian-Pacific region such as China, Singapore and Japan [6–9]. The first case of HFMD was found in New Zealand, and it was officially named HFMD in 1956. In 2008, China listed HFMD as a class C infectious disease. Later, in 2010, coxsackievirus-A6 and coxsackievirus-A10 were identified as the common serotypes of HFMD and have remained so until now [10].

There were gender differences in the incidence of HFMD, with girls slightly lower than boys [11]. The prevalence of HFMD also varies with season, weather, countries and other factors [12]. The incidence of HFMD is relatively high in tropical and temperate zones [13]. The majority of HFMD outbreaks are reported in some Asian-Pacific region countries such as China, India, Malaysia [14]. Since 2014, China HFMD diseases accounted for 87%(9.8million/11.3million) of all HFMD cases reported to WHO [15]. By the end of 2015, China had reported more than 13 million cases of HFMD. The incidence of HFMD, foot and mouth disease (HFMD) had obvious seasonal, with a high incidence in May and June in China. Some severe cases of HFMD even show symptoms of pulmonary edema, pulmonary hemorrhage, nervous system diseases and other severe complications. HFMD remains a major challenge to the development of public health in China. In this study, we aimed to quantitatively analyze the epidemiology, routine laboratory diagnosis, clinical signs and risk factors for HFMD.

## Methods

The Preferred Reporting Items for Systematic Review and Meta-Analyses (PRISMA) statement was used to design and report this review.

### Literature search

A quantitative meta-analysis was conducted to explore the association between epidemiological characteristics, routine laboratory diagnosis, and clinical signs associated with HFMD severity and death. PubMed, Embase, and the Web of Science were comprehensively searched for eligible records (up to April 16, 2021), with no publication time or language restrictions. The search terms were “HFMD” OR “hand, foot and mouth disease”. Then, we performed a manual search of the reference lists from the eligible articles and identified reviews to complete our search.

### Inclusion and exclusion criteria

Two researchers first filtered the retrieved records by accessing the titles and abstracts and then combined them with full-text screening to obtain eligible studies. The research area of this review was all over the world. The inclusion criteria of the literature were as follows: the studies were peer-reviewed for publication; the study time was specified in the study; the subjects were laboratory-confirmed HFMD patients; and the selected articles also met one or both of the following criteria: (1) the studies mentioned the association between the risk factors for and the death from HFMD; and (2) the studies included the clinical signs or routine laboratory diagnosis (including severe and mild disease patient groups) of HFMD.

The exclusion criteria of the studies were as follows: (1) abstract-only articles, letters, editorials, systematic reviews, duplicated publications, and so on; (2) data and information in the article were incomplete, suspicious, or inconsistent; (3) the full text could not be accessed; and (4) overlapping datasets (when facing studies with overlapping data, researchers selected the one with the largest sample size as a priority).

### Data extraction and quality evaluation

Two reviewers completed data extraction and article screening, and disagreements were resolved by discussion and consensus. For all final included articles, we extracted the following data: the first author; year of publication; study region; study period; patient sex; median or mean age; clinical information about patients with HFMD (stiff neck, convulsions, hypersomnia, vomiting, limb shaking, hyperarousal, fever, breathlessness); routine laboratory parameters(white blood cell (WBC) count, C-reactive protein (CRP) levels, interleukin-6 (IL-6) levels, interleukin-10(IL-10) levels, lymphocyte ratio, neutrophil ratio, cluster of differentiation 4 (CD4^+^)); and risk factors. In addition, if the data were not reliable, we imported “NA”.

Moreover, the Study Quality Assessment Tools, which are provided by the National Institute of Health [16], were used to evaluate the quality of the original articles. The evaluation criteria were divided into three levels: poor, fair and good. Based on those criteria, the quality of the studies could be rated.

### Statistical analyses

For the studies reporting the mean and standard deviation (SD) for extracted variables, the pooled estimates of the standard mean difference (SMD) and 95% confidence intervals (CIs) were calculated with a random effect model. When SD was not reported, we estimated the standard deviations based on possible CIs or other variance measures. For the studies reporting specific data on each group of cases, the pooled odds ratio (OR) and 95% CIs were calculated to evaluate the association between those factors and HFMD. The robustness of the results was evaluated by sensitivity analysis which omitted one study at a time. The heterogeneity of the included studies was reflected by the I-squared (I^2^) and chi-square tests. If *p*<0.05 and I^2^ >50%, the heterogeneity was confirmed to be significant. If the heterogeneity was significant, a random effect model was used. Otherwise, a fixed effect model was applied. In addition, Begg’s funnel plot Egger’s test and the trim-and-fill method were used to assess publication bias [17]. All of the statistical analyses were performed by R, version 4.04 meta package.

## Results

### Systematic review

**Fig 1:**
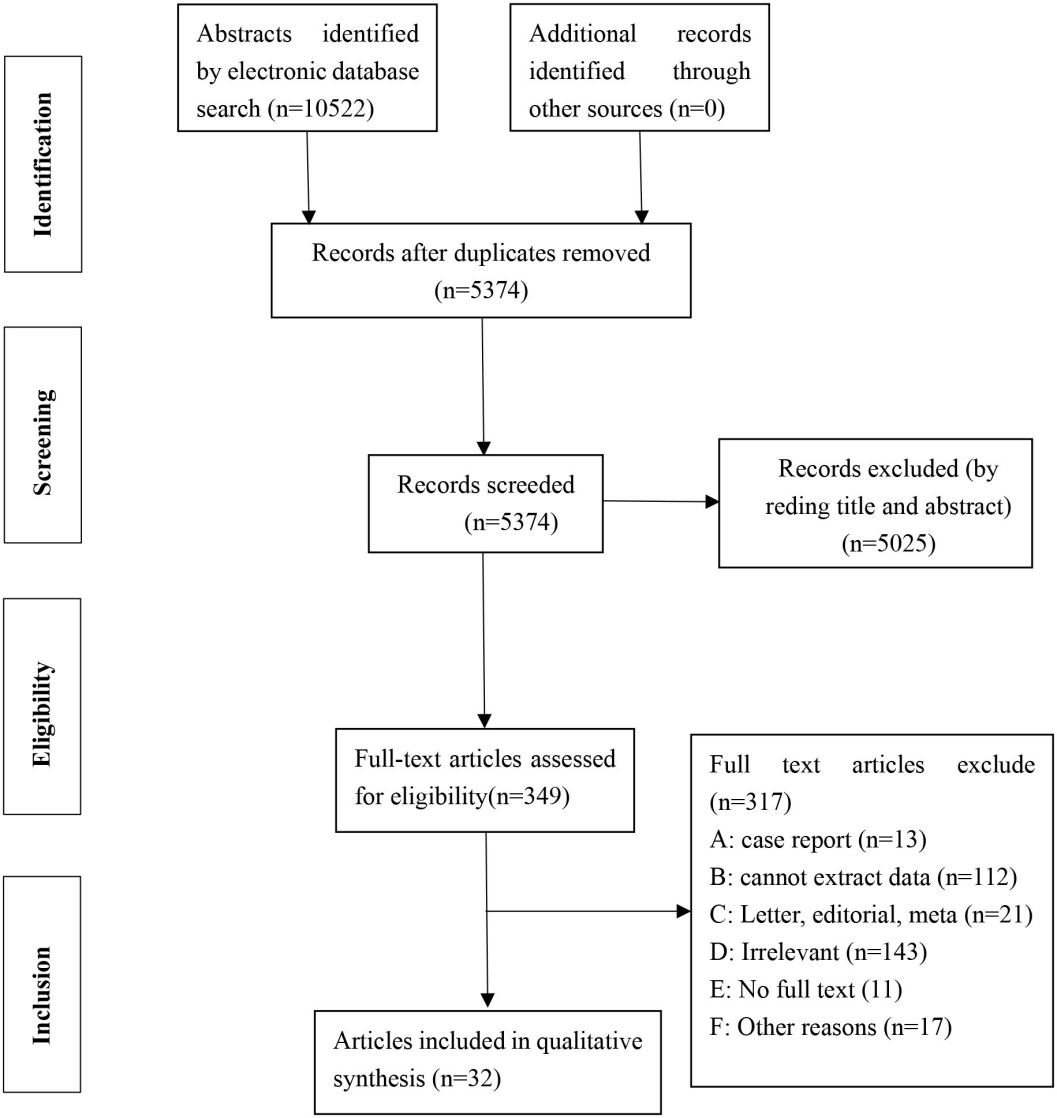
PRISMA flowchart used in the Study selection.

After a preliminary screening of the 10522 identified articles, 5152 articles were removed because they were duplicated. A total of 349 articles were selected to have the full- text reviewed, of which 317 studies were ruled out for the reasons formulated. Finally, we included 32 articles for further data extraction and meta-analysis (Fig 1 and Table 1). Almost all of the included articles reporting cases were from China, with only one article from Singapore. A total of 31 articles reporting cases from China were collected, which included East China (14 articles), Central China (9 articles), West China (5 articles), and Northeast China (1 article), and had steps over multiple regions (2 articles). Geographical or time trends were considered important reasons for publication bias when explaining the evidence for HFMD.

**Table 1:** The basic characteristics of studies in meta-analysis.

First author	Publication year	Region	Year of admitted patients	Case number			Male number	Quality rating
				Total	Mild	Severe		
Chen et al. [18]	2015	East, China	2013–2014	263	142	121	137	Fair
Chen et al. [19]	2013	East, China	2012	126	64	62	80	Fair
Chong et al. [20]	2003	Singapore	2000–2001	138	NA	NA	81	Good
Deng et al. [21]	2016	West, China	2014–2015	216	88	128	133	Fair
Han et al. [22]	2016	East, China	2013–2015	300	150	150	174	Fair
Han et al. [23]	2014	China	2008–2010	91	32	59	55	Good
Han et al. [24]	2011	East, China	2009	102	77	25	62	Good
He et al. [25]	2019	East, China	2012–2014	132	57	75	87	Fair
Huang et al. [26]	2012	East, China	2011	56	45	11	36	Fair
Jiang et al. [27]	2013	Central, China	2011	89	55	34	NA	Fair
Jiang et al. [28]	2012	East, China	2010	573	77	496	375	Fair
Li et al. [29]	2014	Central, China	2012	503	423	80	287	Good
Li et al. [30]	2014	East, China	2012	571	221	350	351	Good
Li et al. [31]	2013	West, China	2011	318	202	116	211	Good
Liu et al. [32]	2013	East, China	2009–2011	164	NA	123	130	Good
Long et al. [33]	2016	Central, China	2011–2014	553	NA	NA	335	Fair
Pan et al. [34]	2012	Central, China	2008–2009	369	140	229	241	Good
Pan et al. [35]	2019	West, China	2017–2018	55	17	38	38	Fair
Qiu et al. [36]	2019	Central, China	2013–2017	7203	NA	NA	4580	Good
Ren et al. [37]	2016	East, China	2015	86	19	67	52	Good
Song et al. [38]	2014	Central, China	2010–2012	167	NA	NA	107	Good
Tang et al. [39]	2011	East, China	2008–2009	186	62	124	120	Good
Wang et al. [40]	2014	Central, China	2009	120	60	60	72	Fair
Wang et al. [41]	2020	West, China	2013–2018	459	NA	NA	295	Good
Xu et al. [42]	2011	Central, China	2010	315	NA	210	210	Good
Yang et al. [43]	2020	East, China	2017	261	206	55	174	Good
Zhang et al. [44]	2017	East, China	2009–2014	530	345	185	379	Good
Zhang et al. [45]	2011	China	2008–2009	765220	NA	4067	NA	Fair
Zhang et al. [46]	2016	Central, China	2014–2015	100	58	42	64	Good
Zheng et al. [47]	2017	West, China	2009–2016	179	NA	52	112	Good
Zheng et al. [48]	2017	East, China	2014–2015	82	30	52	44	Good
Zhou et al. [49]	2012	Northeast, China	2008–2011	2379	1798	581	1385	Good

NA: not applicable

### Clinical symptoms of HFMD

**Fig 2:**
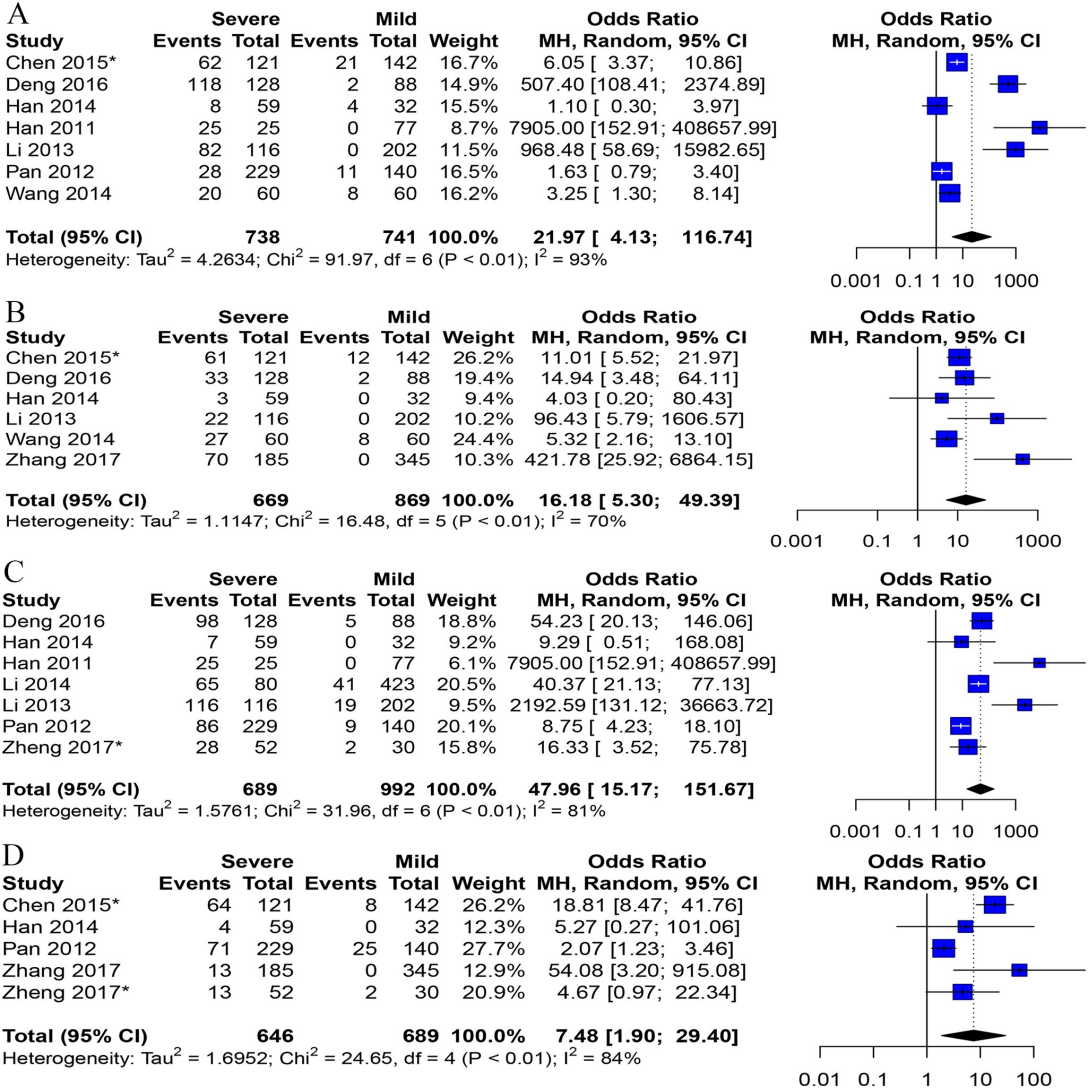
Forest plots of meta-analysis on a panel of clinical signs. A Hypersomnia, B convulsion, C Limb shaking, D Breathlessness. *Data is converted from the original data.

A total of 13 studies involving 3,173 patients assessed the relationship between HFMD and clinical symptoms. We analyzed the association between clinical symptoms and the severity of HFMD, and the results were as follows: hypersomnia (7 studies, 95% CI: 4.13 to 116.74), convulsion (6 studies, 95% CI: 5.30 to 49.39), vomiting (11 studies, 95% CI: 3.49 to 11.44), limb shaking (7 studies, 95% CI: 15.17 to 151.67), fever (8 studies, 95% CI: 2.87 to 12.06), breathlessness (5 studies, 95% CI: 1.90 to 29.40), hyperarousal (3 studies, 95% CI: 4.75 to 285.46), and stiff neck (3 studies, 95% CI: 1.76 to 110.28). For each symptom considered in three or more studies, the odds ratio (OR), 95% confidence interval (95% CI) and p value are shown in Fig 2 and S1 Table. There was significant heterogeneity (P < 0.1) among studies meta-analyzed of the 8 clinical symptoms, which used a random effect model with I-squared values >50%.

### Routine laboratory diagnosis of HFMD

**Fig 3:**
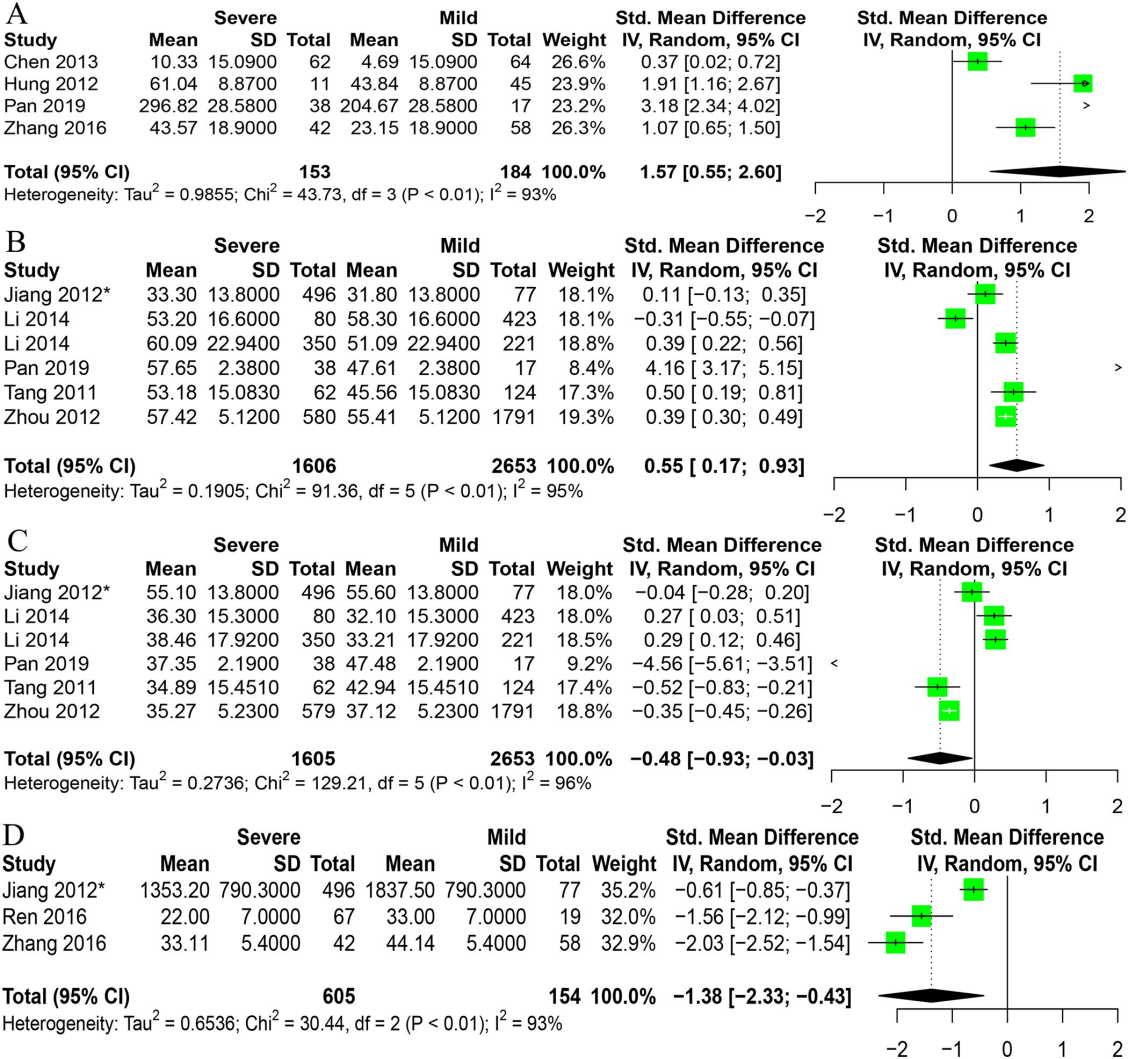
Forest plots of meta-analysis on a panel of routine laboratory parameters. A IL-6, B Neutrophils, C Lymphocytes, D CD4+. *Data is converted from the original data.

Sixteen studies, including 6048 cases, analyzed the connection between HFMD and routine laboratory diagnosis. We analyzed the routine laboratory diagnosis association between mild and severe HFMD. As shown in Fig 3 and S1 Table, the results of routine laboratory diagnoses for patients with severe HFMD showed IL-6 levels (SMD = 1.57, 95%CI: 0.55 to 2.60), and neutrophils ratio (SMD = 0.55, 95%CI: 0.17 to 0.93) compared with patients with mild HFMD, but the results displayed reduced CD4^+^ levels (SMD = -1.38, 95%CI: -2.33 to -0.43), lymphocytes ratio (SMD = -0.48, 95%CI: -0.93 to -0.33) and WBC count (SMD = -0.68, 95%CI: -1.33 to -0.04). However, there was no significant association with IL-10 and CRP (SMD = 0.33, 95%CI: -0.96, 1.62) levels in patients with severe cases of HFMD compared with patients with mild cases.

### Risk factors for HFMD

**Fig 4:**
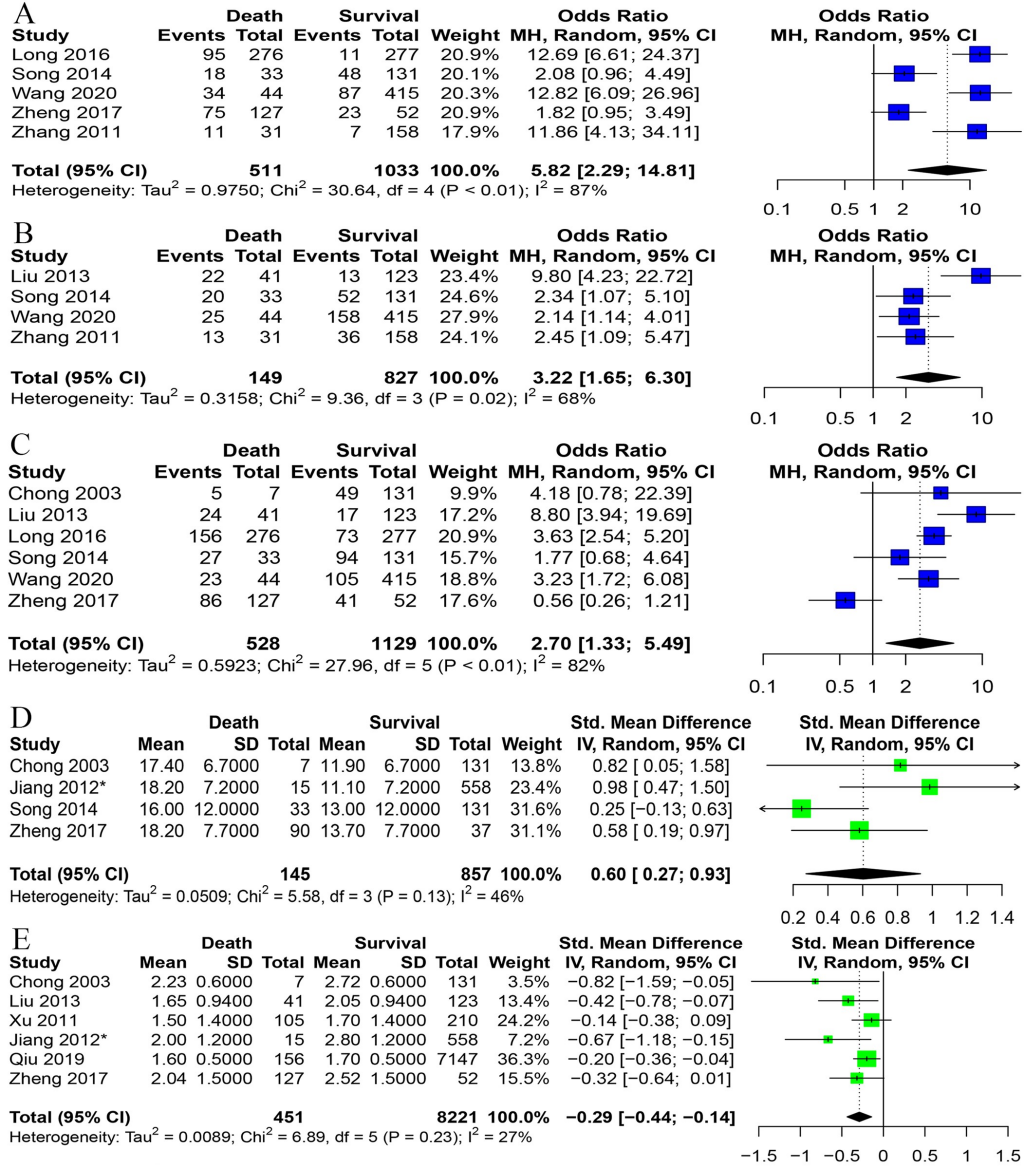
Forest plots on a panel of risk factors. A Cyanosis, B Fast heart rate, C Vomiting, D WBC count, E Age. *Data is converted from the original data.

For the risk of death from HFMD, we analyzed sex, age, cyanosis, fast heart rate, vomiting, duration of fever ≥3 days, and WBC count. Cyanosis (OR = 5.82, 95% CI: 2.29 to 14.81), fast heart rate (OR = 3.22, 95% CI: 1.65 to 6.30), and vomiting (OR = 2.70, 95% CI: 1.33 to 5.49) were risk factors for death in HFMD patients. A duration of fever ≥3 days and male sex had no significant risk of death from the disease. The WBC count (SMD = 0.60, 95% CI: 0.27 to 0.93) was elevated in patients who died compared with those who survived. Patient age was lower in patients who died compared with those who survived (SMD = -0.29, 95% CI: -0.44 to -0.14) (Fig 4 and S1 Table).

### Sensitivity analysis and publication bias

Sensitivity analysis that omitted one study at a time shows that the results of our analysis are robust (S1–S3 Figs). The heterogeneity between the studies varied substantially. Publication bias, measured by Begg’s and Egger’s tests, was evident in only three analyses. However, these two tests may lack the statistical power to detect bias when the number of studies is small (i.e., fewer than 10), as we only included 3–8 studies.

## Discussion

Children are most vulnerable to HFMD, even though most cases can be treated effectively. However, there are still some severe cases that progress rapidly and die in a short time from cardiopulmonary failure [8, 50]. With the issues related to an aging population, some countries have introduced corresponding policies to alleviate the problem of population aging, such as the introduction of the three-child policy, which makes the susceptible population to HFMD gradually increase. In our review, 32 articles and a total of 781903 HFMD cases in the region of China and Singapore were included.

All of the factors that we analyzed in the review significantly increased the risk of poor prognosis in patients with HFMD. The earlier the identification of the factors we analyzed occurred, the better the intensification of HFMD was avoided. Therefore, doctors need to know these factors to reduce severe cases of HFMD. Our review showed that all of the clinical symptoms had significant heterogeneity and related with HFMD, and a random effects model was used. China has published diagnosis and treatment guidelines for HFMD [51], which included earlier identification of severe patients. Some of our analyzed clinical signs were included in the guidelines, while others were not. A relevant meta-analysis involving 19 separate studies found that clinical characteristics such as convulsion, vomiting, limb shaking and fever were significantly increased risk of severe HFMD which is consistent with our results [12]. In addition, previous studies on hypersomnia have same conclusion: thought that hypersomnia have increased risk of severe HFMD [52]. For these reasons, we should increase the detection of some earlier diagnosis clinical signs that were not included in guidelines, such as stiff neck, hyperarousal and hypersomnia.

In terms of the routine laboratory parameters of HFMD, the Chinese diagnosis and treatment guidelines included WBC count, which we analyzed in this review. Although we extracted and analyzed the WBC count, the results of the analysis are different from the guidelines, and the lack of literature could have led to this result. Compared to those in patients with mild HFMD, the IL-6 and neutrophil ratio levels were increased in patients with severe HFMD, and the lymphocyte ratio and CD4+ count were reduced. In addition, CRP level was not associated with intensification of HFMD. Therefore, we think that the IL-6 levels, CD4+ levels, neutrophil ratio and lymphocyte ratio could be used as reference indices to identify patients with severe HFMD.

Currently, the risk factors for death from HFMD are not completely clear. Therefore, it is important to identify the risk factors associated with patients who died from HFMD. In this review, we evaluated the risk factors for HFMD in patients. Age is a significant factor that is associated with HFMD morbidity and death. Cyanosis and vomiting were reported in many studies, that suggested both were associated with HFMD mortality and were confirmed in the review [33, 38]. According to our results, a fast heart rate was a risk factor for death from HFMD. The WBC count is increased when patients have a poor prognosis. Wang et al. found that male patients more easily died from HFMD than female patients [53]. However, our study showed that male sex variable was not associated with death from HFMD. Similarly, a duration of fever ≥3 days was not associated with HFMD mortality.

Our study also had some limitations. First, our study did not analyze some variables because these indicators lacked data in the included articles. Second, our study had a certain amount of influence on the quality of the included articles. Third, we included 32 articles in this study, but relatively fewer literatures were included for every index and, at most, 11 articles were included. Fourth, almost all of the included articles were from China, and only one of the articles was from Singapore. When we excluded the article from Singapore, the results of the review were not altered.

## Conclusions

In conclusion, China many regions had an occurrence of HFMD. China was the country with the highest incidence of HFMD. May to June was peak of the epidemic, and low age were a high-risk group of death. According to the results, cyanosis, a fast heart rate, vomiting and an increased WBC count appeared to increase HFMD mortality. We found that twelve factors are associated with the severe HFMD. Previous conclusion showed that convulsion, hypersomnia, vomiting, limb shaking and fever were significantly increased risk of severe HFMD which is consistent with our results. More attention should be paid when patients exhibit hypersomnia, convulsions, stiff neck, vomiting, limb shaking, hyperarousal, fever, breathlessness and certain laboratory parameters (IL-6 levels, CD4+ levels, neutrophil ratio and lymphocyte ratio) which could reduce the intensification of HFMD. In addition, relevant departments should combine epidemiological characteristics, risk factors and clinical signs to formulate policies to control and prevent HFMD.

## Supporting information

S1 Fig Sensitivity analysis that omitting one study at a time of meta-analysis of clinical signs. A Breathlessness, B Convulsion, C Fever, D Hyperarousal, E Hypersomnia, F Limb shaking, G Stiff neck, H Vomiting.
(TIF)

S2 Fig Sensitivity analysis that omitting one study at a time of meta-analysis of routine laboratory parameters. A CD4+, B CRP, C IL-6, D IL-10, E Lymphocytes, F Neutrophils, G WBC count.
(TIF)

S3 Fig Sensitivity analysis that omitting one study at a time of meta-analysis of death risk factors. A Age, B Cyanosis, C Duration of fever ≥ 3 days, D Fast heart rate, E Male, F Vomiting, G WBC count.
(TIF)

S1 Table Main clinical and laboratory parameters hand-foot-and- mouth disease patients in this review. (DOCX)

S2 Table The detail results of quality assessment. (DOCX)

S1 Checklist PRISMA checklist. (DOC)

S1 File (PDF)

S2 File (DOCX)
