# Supplementary figures and images for: Epidemiological characteristics, routine laboratory diagnosis, clinical signs and risk factors for hand, -foot -and -mouth disease: A systematic review and meta-analysis
Source: PLoS One. 2022 Apr 28;17(4):e0267716. doi: 10.1371/journal.pone.0267716 (PMC9049560; doi:10.1371/journal.pone.0267716)

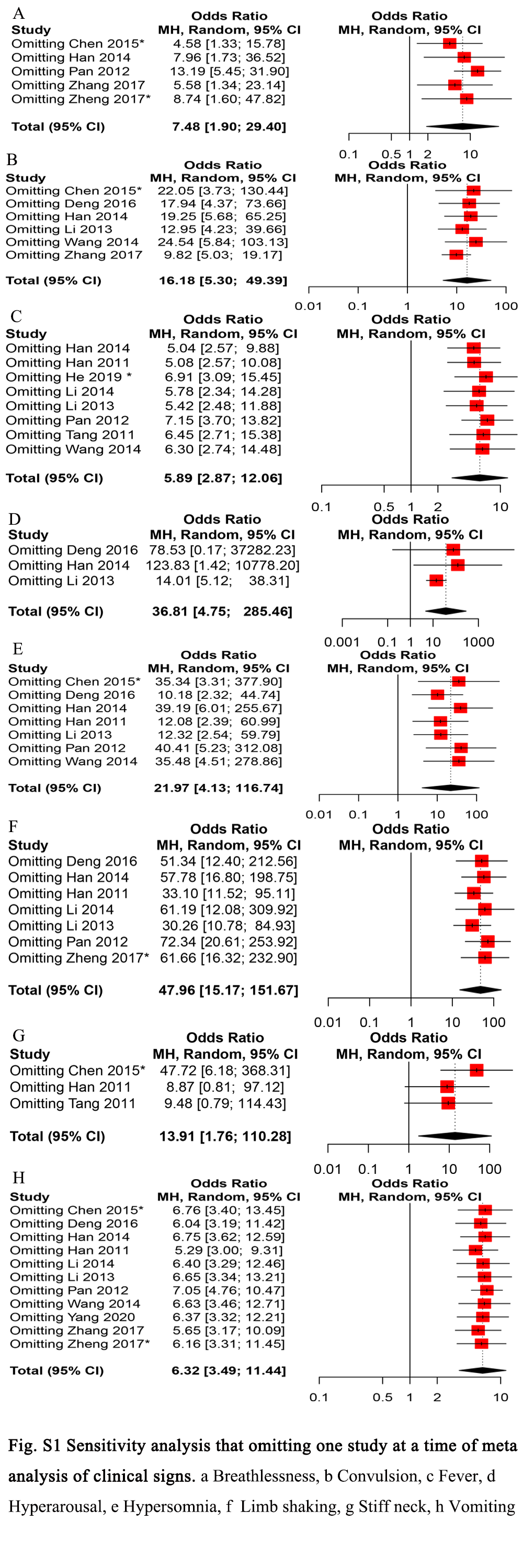

Supplement: S1 Fig — A Breathlessness, B Convulsion, C Fever, D Hyperarousal, E Hypersomnia, F Limb shaking, G Stiff neck, H Vomiting. (TIF) [file pone.0267716.s001.tif]

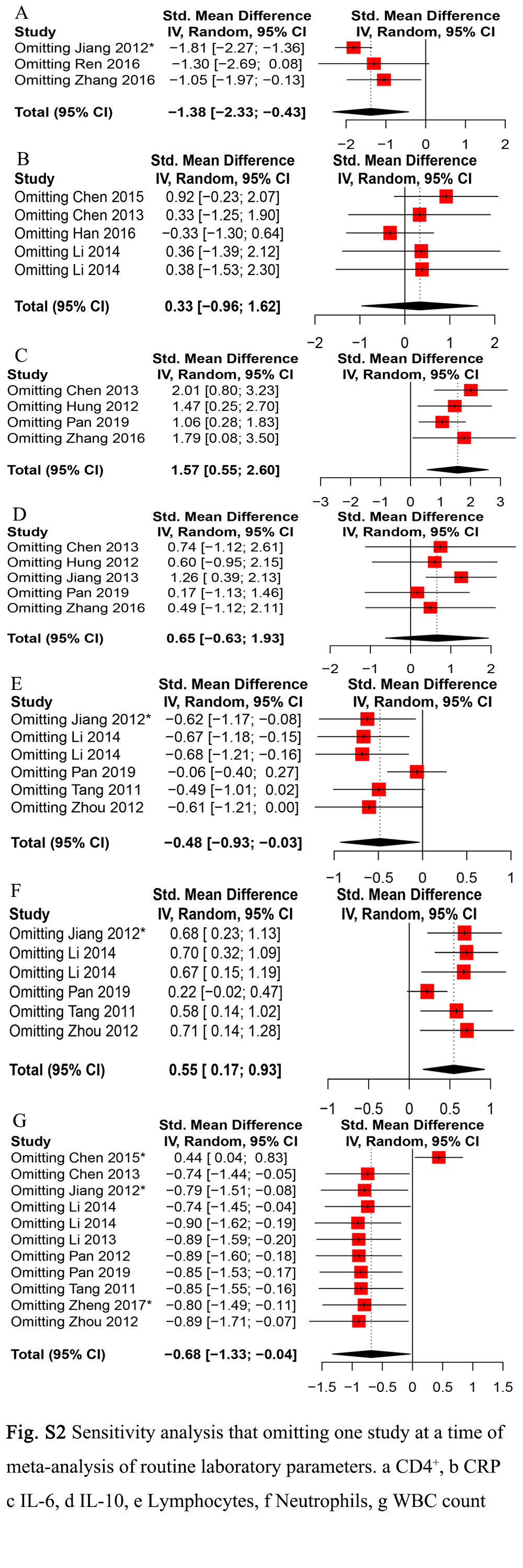

Supplement: S2 Fig — A CD4+, B CRP, C IL-6, D IL-10, E Lymphocytes, F Neutrophils, G WBC count. (TIF) [file pone.0267716.s002.tif]

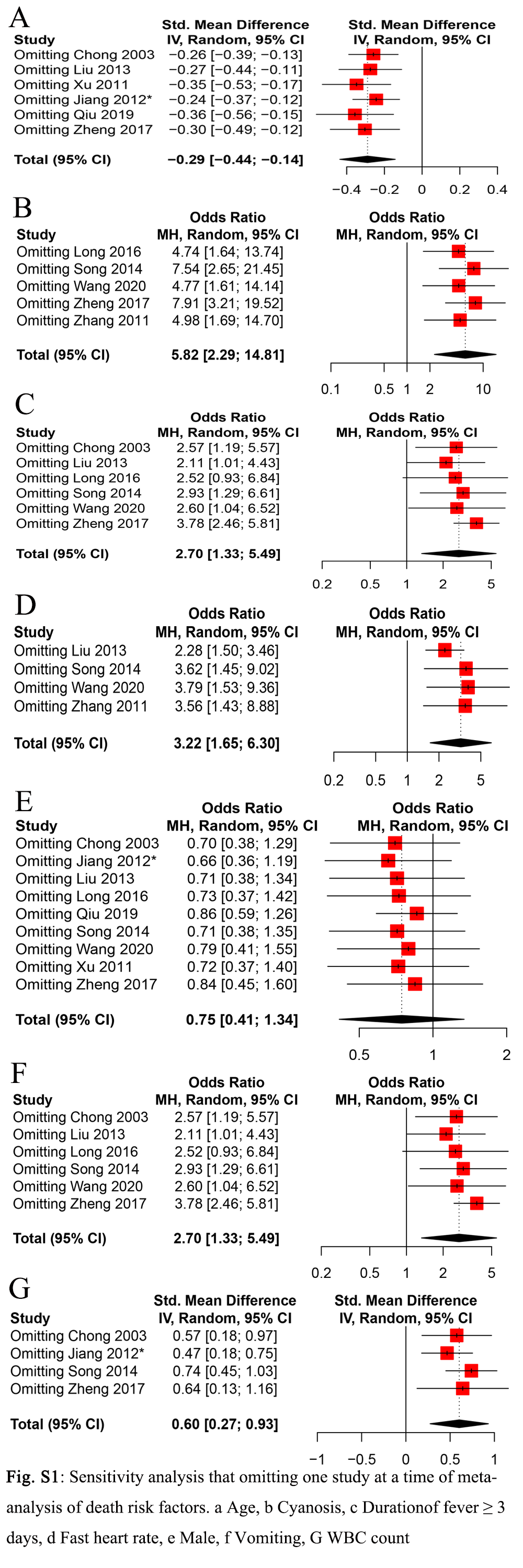

Supplement: S3 Fig — A Age, B Cyanosis, C Duration of fever ≥ 3 days, D Fast heart rate, E Male, F Vomiting, G WBC count. (TIF) [file pone.0267716.s003.tif]
