# Supplementary material for: Epidemiological characteristics, routine laboratory diagnosis, clinical signs and risk factors for hand, -foot -and -mouth disease: A systematic review and meta-analysis
Source: PLoS One. 2022 Apr 28;17(4):e0267716. doi: 10.1371/journal.pone.0267716 (PMC9049560; doi:10.1371/journal.pone.0267716)
Supplement: S1 Table — (DOCX) [file pone.0267716.s004.docx]

S1 Table Main clinical and laboratory parameters hand-foot-and- mouth disease patients in this review

|  | Number of studies | Number of events/Number of severe | Number of events/Number of non-severe | OR [95% CI] | p-value | T^2^ | I^2^ | Begg’s test  **(p-value)** | Egg’s test  (p-value) |
| --- | --- | --- | --- | --- | --- | --- | --- | --- | --- |
|  |  |  |  |  |  |  |  |  |  |
| Severe (case) vs non-severe HFMD (controls) | | | | | | | | | |
| **Symptoms and signs** | | | | | | | | | |
| Hypersomnia | 7 | 343/738 | 46/741 | 21.97[4.13 - 116.74] | <0.01 | 4.2634 | 93% | P = 0.2931 | P = 0.1089 |
| Convulsions | 6 | 216/669 | 22/869 | 16.18[5.30 – 49.39] | <0.01 | 1.1147 | 70% | P = 0.5073 | P = 0.2345 |
| Stiff neck | 3 | 44/208 | 11/343 | 13.91[1.76 – 110.28] | 0.06 | 2.1568 | 64% | P = 0.1172 | P = 0.0117 |
| Vomiting | 11 | 435/1110 | 175/1745 | 6.32[3.49 – 11.44] | <0.01 | 0.7279 | 83% | P = 0.2429 | P = 0.0530 |
| Limb shaking | 7 | 425/689 | 76/992 | 47.76[15.17–151.67] | <0.01 | 1.5761 | 81% | P = 0.6523 | P = 0.2504 |
| Hyperarousal | 3 | 286/303 | 90/322 | 36.81[4.75 – 285.46] | <0.01 | 2.6267 | 87% | P = 0.6015 | P = 0.3994 |
| Fever | 8 | 636/708 | 612/1115 | 5.89[2.87 – 12.06] | <0.01 | 0.6182 | 67% | P =0.1376 | P = 0.5776 |
| Breathlessness | 5 | 165/646 | 35/689 | 7.48[1.90 – 29.40] | <0.01 | 1.6952 | 84% | P = 0.6242 | P = 0.4178 |
| **Laboratory parameter** | **Number of studies** | **Number of severe** | **Number of non-severe** | **SMD [95% CI]** | **p-value** | **T^2^** | **I^2^** | **Begg’s test**  **(p-value)** | **Egger test**  **(p-value)** |
| WBC count | 11 | 2183 | 3123 | -0.68[-0.33 – (-0.04) | <0.01 | 1.1298 | 99% | P = 0.3918 | P = 0.5592 |
| CRP | 5 | 763 | 1000 | 0.33[-0.96 – 1.62] | <0.01 | 2.1479 | 99% | P = 0.3272 | P = 0.7922 |
| IL-6 | 4 | 153 | 184 | 1.57[0.55 – 2.60] | <0.01 | 0.9855 | 93% | P = 0.0415 | P = 0.0465 |
| IL-10 | 5 | 187 | 239 | 0.65[-0.63 – 1.93] | <0.01 | 2.0488 | 97% | P = 0.3272 | P = 0.6589 |
| Lymphocytes | 6 | 1605 | 2653 | -0.48[-0.93 – (-0.03)] | <0.01 | 0.2736 | 96% | P = 0.1885 | P = 0.6461 |
| Neutrophils | 6 | 1606 | 2653 | 0.55[0.17 – 0.93] | <0.01 | 0.1905 | 95% | P = 0.1885 | P = 0.6461 |
| CD4+ | 3 | 605 | 154 | -1.38[-2.33 – (-0.43)] | <0.01 | 0.6536 | 93% | P = 0.6015 | P = 0.2415 |
|  | **Number of studies** | **Number of non-survivors** | **Number of survivors** | **SMD [95% CI]** | **p-value** | **T^2^** | **I^2^** | **Begg’s test**  **(p-value)** | **Egg’s test (p-value)** |
| Non-survivors (cases) vs survivors (controls) | | | | | | | | | |
| WBC count | 4 | 145 | 857 | 0.60[0.27 – 0.93] | 0.13 | 0.0509 | 46% | P = 0.1742 | P = 0.3618 |
| Age | 6 | 451 | 8221 | -0.29[-0.44 – (-0.14)] | 0.23 | 0.0089 | 27% | P = 0.0388 | P = 0.0127 |
|  | **Number of studies** | **Number of events/Number of non-survivors** | **Number of events/Number of survivors** | **OR [95% CI]** | **p-value** | **T^2^** | **I^2^** | **Begg’s test**  **(p-value)** | **Egg’s test (p-value)** |
| Male | 9 | 441/804 | 5784/9044 | 0.55[0.41 – 1.34] | <0.01 | 0.6265 | 87% | P = 1.000 | P = 0.3330 |
| Cyanosis | 5 | 233/511 | 176/1033 | 5.82[2.29 – 14.81] | <0.01 | 0.9750 | 87% | P = 1.000 | P = 0.7132 |
| Fast heart rate | 4 | 80/149 | 259/827 | 3.22[1.65 – 6.30] | 0.02 | 0.3158 | 68% | P = 0.0415 | P = 0.3798 |
| Vomiting | 6 | 321/528 | 379/1129 | 2.70[1.33 – 5.49] | <0.01 | 0.5923 | 82% | P = 0.8510 | P = 0.7072 |
| Duration of fever ≥ 3days | 3 | 257/353 | 515/823 | 1.18[0.52 – 2.69] | <0.01 | 0.4165 | 80% | P = 0.6015 | P = 0.6415 |
