# Supplementary material for: Epidemiological characteristics, routine laboratory diagnosis, clinical signs and risk factors for hand, -foot -and -mouth disease: A systematic review and meta-analysis
Source: PLoS One. 2022 Apr 28;17(4):e0267716. doi: 10.1371/journal.pone.0267716 (PMC9049560; doi:10.1371/journal.pone.0267716)
Supplement: S2 Table — (DOCX) [file pone.0267716.s005.docx]

S2_Table The detail results of quality assessment

| First author | Publication year | The research type | Conform to the point | score | Quality rating |
| --- | --- | --- | --- | --- | --- |
| Chen et al[20] | 2015 | Case Series Study | 1,2,4,6,8,9 | 6 | Fair |
| Chen et al[21] | 2013 | Case-control study | 1,2,4,5,6,9,10,11 | 8 | Fair |
| Chong et al[22] | 2003 | Case Series Study | 1,2,3,4,6,8,9 | 7 | Good |
| Deng et al[23] | 2016 | Case series study | 1,2,4,6,8,9 | 6 | Fair |
| Han et al[24] | 2016 | Case Series Study | 1,2,4,6,8,9 | 6 | Fair |
| Han et al[25] | 2014 | Case Series Study | 1,2,3,4,5,6,8,9 | 8 | Good |
| Han et al[26] | 2011 | Case Series Study | 1,2,3,4,5,6,8,9 | 8 | Good |
| He et al[27] | 2019 | Case-control study | 1,2,4,5,6,8,10,12 | 8 | Fair |
| Huang et al[28] | 2012 | Case-control study | 1,2,4,6,10 | 5 | Fair |
| Jiang et al[29] | 2013 | Case-control study | 1,2,4,5,6,10 | 6 | Fair |
| Jiang et al[30] | 2012 | Case Series Study | 1,2,3,4,5,6,8,9 | 8 | Fair |
| Li et al[31] | 2014 | Case Series Study | 1,2,3,4,6,8,9, | 7 | Good |
| Li et al[32] | 2014 | Case Series Study | 1.2.3.4.6.8.9 | 7 | Good |
| Li et al[33] | 2013 | Case Series Study | 1,2,3,4,6,8,9 | 7 | Good |
| Liu et al[34] | 2013 | Case Series Study | 1,2,3,4,6,8,9, | 7 | Good |
| Long et al[35] | 2016 | Case-control study | 1,2,3,4,5,6,7,12 | 8 | Fair |
| Pan et al[36] | 2012 | Case Series Study | 1,2,3,4,6,8,9 | 7 | Good |
| Pan et al[37] | 2019 | Case-control study | 1,2,4,6,10,11 | 6 | Fair |
| Qiu et al[38] | 2019 | Case-control study | 1,2,3,4,7,8,9,10,11,12 | 10 | Good |
| Ren et al[39] | 2016 | Case Series Study | 1,2,3,4,6,8,9 | 7 | Good |
| Song et al[40] | 2014 | Case Series Study | 1,2,3,4,6,8,9 | 7 | Good |
| Tang et al[41] | 2011 | Case-control study | 1,2,4,5,6,8,9,10,11,12 | 10 | Good |
| Wang et al[42] | 2014 | Case-control study | 1,2,4,5,6,10 | 6 | Fair |
| Wang et al[43] | 2020 | Case-control study | 1,2,3,4,7,8,9,10,12 | 9 | Good |
| Xu et al[44] | 2011 | Case series study | 1,2,3,4,6,8,9 | 7 | Good |
| Yang et al[45] | 2020 | Case series study | 1,2,3,4,6,8,9 | 7 | Good |
| Zhang et al[46] | 2017 | Case series study | 1,2,3,4,6,8,9 | 7 | Good |
| Zhang et al[47] | 2011 | Cohort study | 1,2,3,4,5,7,11,12,13,14 | 10 | Fair |
| Zhang et al[48] | 2016 | Case-control study | 1,2,3,4,5,6,7,12 | 8 | Good |
| Zheng et al[49] | 2017 | Case series study | 1,2,3,4,6,8,9 | 7 | Good |
| Zheng et al[50] | 2017 | Case series study | 1,2,3,4,6,8,9 | 7 | Good |
| Zhou et al[51] | 2012 | Case series study | 1,2,3,4,5,6,8,9 | 8 | Good |
