# Supplementary material for: Epidemiological characteristics, routine laboratory diagnosis, clinical signs and risk factors for hand, -foot -and -mouth disease: A systematic review and meta-analysis
Source: PLoS One. 2022 Apr 28;17(4):e0267716. doi: 10.1371/journal.pone.0267716 (PMC9049560; doi:10.1371/journal.pone.0267716)
Supplement: S1 File — (PDF) [file pone.0267716.s007.pdf]

STATEMENT OF DELIVERY

Invoice# SV2MTYFRB

Balance Due

CNY0.00

601 W Main St, Ste 102
Durham, North Carolina, 27701, USA
Tax ID: 412141424

Delivery Date :	16 Aug 2021
Submission :	V2MTYFRB
Word Count :	2404
Title :	Epidemiology, routine laboratory diagnosis, clinical signs and risk factors with Hand-foot-and-mout…

Bill To

志杰 易
15837189100
李懿
河南省郑州市郑东新区农业南路105号
河南省疾病预防控制中心
郑州市
450016
China

#	Item & Description	Base Price	Amount
1	Standard Editing<br>Standard Editing	1,604.75	1,604.75

Sub Total	1,604.75
**Total**	**CNY1,604.75**
Payment Made	(-) 1,604.75
**Balance Due**	**CNY0.00**

请注意：本电子收据创建时间显示的是美国东部时间，该时间比您的当地时间可能晚12个（春夏）或13个小时（秋冬）。
