# Supplementary material for: Epidemiological characteristics, routine laboratory diagnosis, clinical signs and risk factors for hand, -foot -and -mouth disease: A systematic review and meta-analysis
Source: PLoS One. 2022 Apr 28;17(4):e0267716. doi: 10.1371/journal.pone.0267716 (PMC9049560; doi:10.1371/journal.pone.0267716)

Take Pubmed as an example, here are our detailed search steps.

First step: click on advanced in Pubmed.


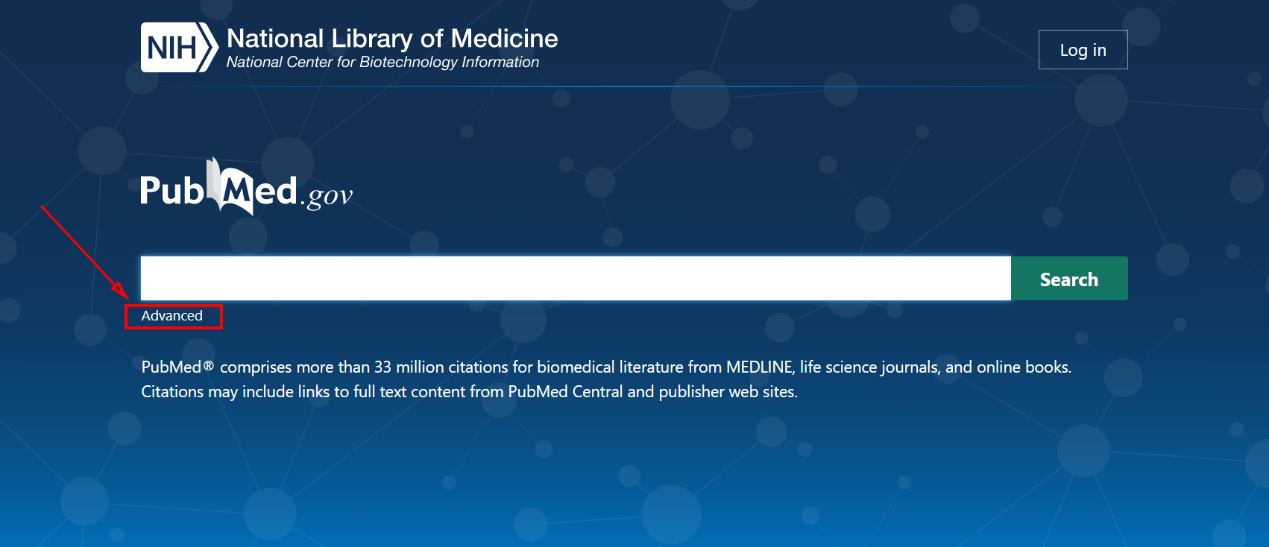


Second step: Enter term here.


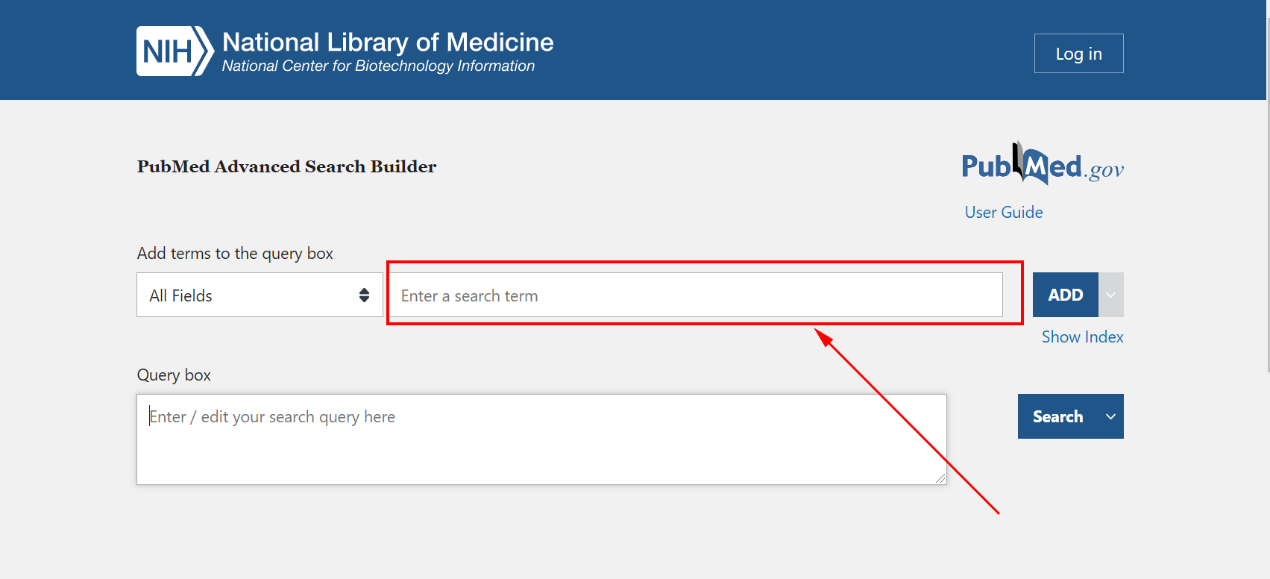


Third step: Input HFMD and click ADD.


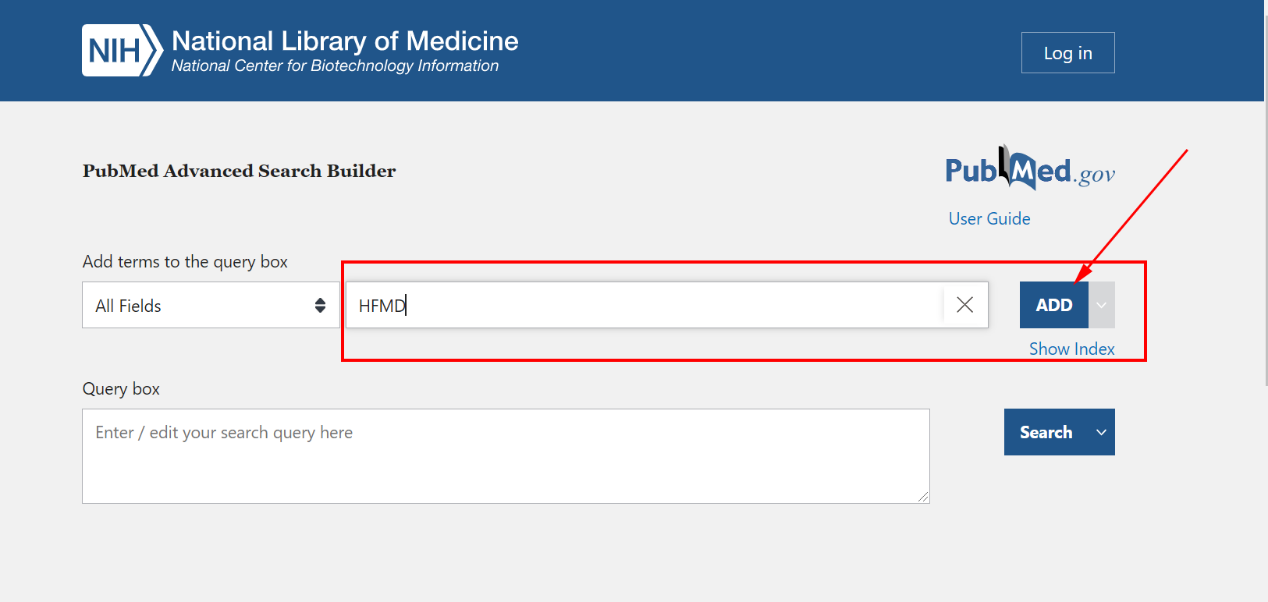


Fourth step: Input hand foot and mouth disease and click AND.


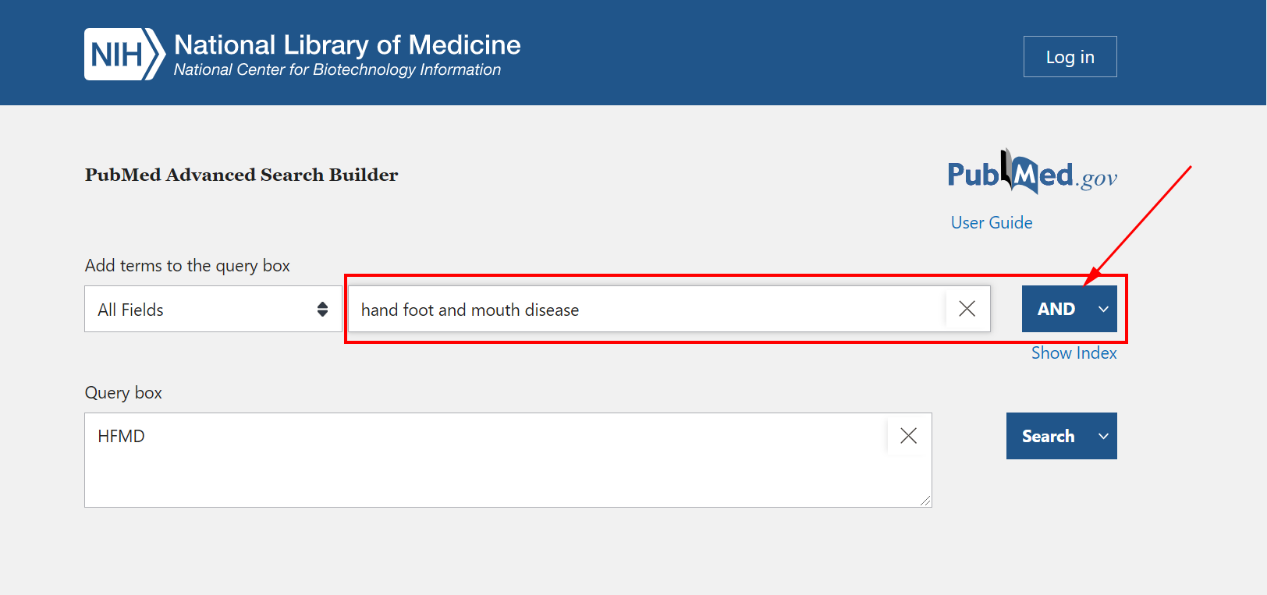


Fifth step: The result is obtained in the query box. Click Search. This study was searched in PubMed in April 16, 2021.


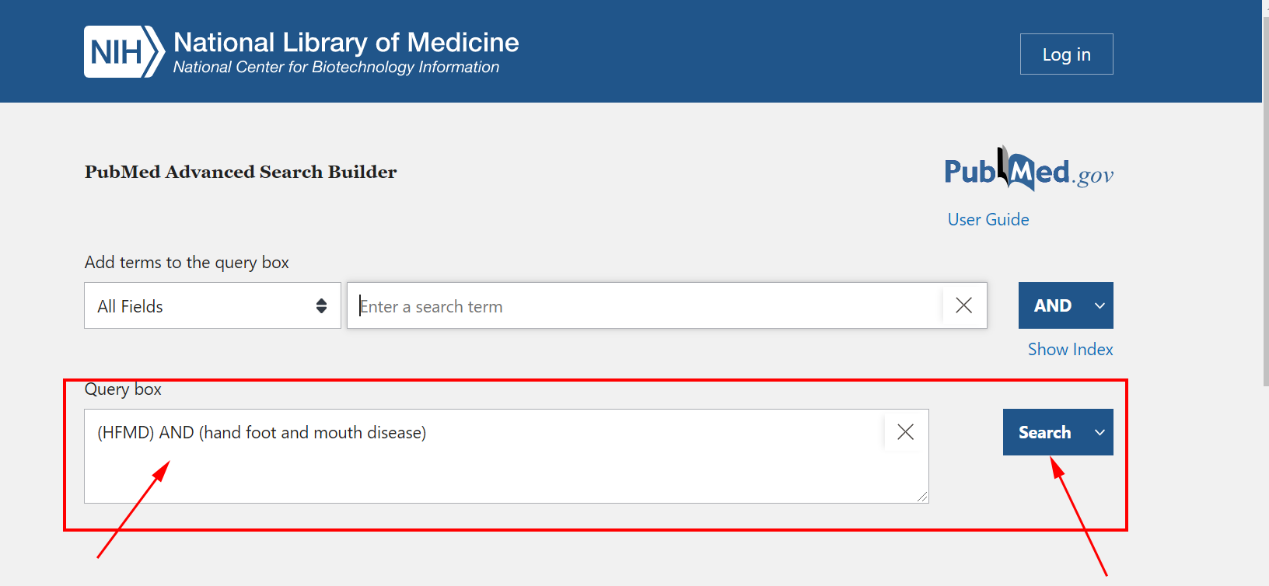

Supplement: S2 File — (DOCX) [file pone.0267716.s008.docx]
